# Protein Supplementation During or Following a Marathon Run Influences Post-Exercise Recovery

**DOI:** 10.3390/nu10030333

**Published:** 2018-03-10

**Authors:** Michael J. Saunders, Nicholas D. Luden, Cash R. DeWitt, Melinda C. Gross, Amanda Dillon Rios

**Affiliations:** Department of Kinesiology, James Madison University, Harrisonburg, VA 22807, USA; ludennd@jmu.edu (N.D.L.); Cashrdewitt@gmail.com (C.R.D.); mgross624@gmail.com (M.C.G.); dillo2am@gmail.com (A.D.R.)

**Keywords:** carbohydrate, protein, post-exercise recovery, sports nutrition

## Abstract

The effects of protein supplementation on the ratings of energy/fatigue, muscle soreness [ascending (A) and descending (D) stairs], and serum creatine kinase levels following a marathon run were examined. Variables were compared between recreational male and female runners ingesting carbohydrate + protein (CP) during the run (CP_During_, *n* = 8) versus those that were consuming carbohydrate (CHO_During,_ *n* = 8). In a second study, outcomes were compared between subjects who consumed CP or CHO immediately following exercise [CP_Post_ (*n* = 4) versus CHO_Post_ (*n* = 4)]. Magnitude-based inferences revealed no meaningful differences between treatments 24 h post-marathon. At 72 h, recovery [Δ(_72 hr-Pre_)] was likely improved with CP_During_ versus CHO_During_, respectively, for Physical Energy (+14 ± 64 vs −74 ± 70 mm), Mental Fatigue (−52 ± 59 vs +1 ± 11 mm), and Soreness_-D_ (+15 ± 9 vs +21 ± 70 mm). In addition, recovery at 72 h was likely-very likely improved with CP_Post_ versus CHO_Post_ for Physical Fatigue, Mental Energy, and Soreness_-A_. Thus, protein supplementation did not meaningfully alter recovery during the initial 24 h following a marathon. However, ratings of energy/fatigue and muscle soreness were improved over 72 h when CP was consumed during exercise, or immediately following the marathon.

## 1. Introduction

Numerous studies have reported that the co-ingestion of carbohydrate and protein (CP) has positive effects on post-exercise recovery in endurance athletes, in comparison to carbohydrate alone (CHO). Specifically, supplemental protein has been associated with reduced post-exercise muscle soreness [1,2,3,4], attenuated biomarkers of sarcolemmal disruption (i.e., creatine kinase; CK [2,4,5,6,7,8,9] and myoglobin; Mb [9,10]), enhanced mood/energy states [3,11,12], recovery of muscle function [7,9,12], and subsequent exercise performance [6,10,13]. However, these findings have not been unanimous, and other studies have reported no effects of CP on these variables post-exercise (i.e., [14,15,16]). The reasons for these discrepancies between studies are not well understood, but are likely related to inter-study differences in experimental factors, such as exercise protocols, subject populations/variance, recovery time, and nutritional interventions [17,18]. Therefore, there is a need to investigate the efficacy of CP in specific sport populations, in order to provide appropriate recommendations for endurance athletes. Towards this end, the effects of CP on post-exercise recovery have been previously investigated in endurance-related athletes following heavy resistance exercise [7], endurance/interval cycling [2,5,9,10,12], combative and team-sport training [3,8,13], and some running events [1,4]. Surprisingly, to our knowledge, no studies have investigated the effects of CP following marathon running. This information is particularly impactful due to significant growth in marathon participation, with the number of marathon finishers in the United States of America (U.S.A.) increasing >125% since 1990, to >500,000 per year in 2013–2016 [19]. In addition, the markers of sarcolemmal disruption and soreness increase with running distance [20], and marathon running is associated with substantial increases in muscle soreness and CK, and functional impairment post-exercise [21,22].

There is also a need for further information regarding the appropriate timing of CP supplementation, particularly in long duration endurance sports in which nutrient consumption is recommended during exercise [23]. The vast majority of studies have investigated the effects of CP that is ingested in the post-exercise time-period. However, Valentine and colleagues [9] reported that CP ingestion during prolonged cycling (with no post-exercise nutritional intervention) attenuated Mb and CK levels, and enhanced muscle function 24 h after exercise, in comparison to CHO. Similarly, Hall and associates [10] reported that when cyclists ingested CP during an initial bout of cycling, post-exercise Mb levels were attenuated, and subsequent time-trial performance (4 h later) was likely to be enhanced, versus when CHO was consumed during exercise. The impact of CP consumed during exercise was particularly notable in this study, as post-exercise CP was consumed in both the CHO and CP trials. However, others have reported that CP that is consumed during prolonged cycling has no significant effects on post-exercise markers of sarcolemmal disruption, soreness ratings, or muscle function [16]. Therefore, further information is needed regarding the effects of CP ingested during endurance exercise, particularly in sporting events other than cycling. Therefore, the primary purposes of the present study were to determine the effects of CP ingestion: (a) during marathon running, and (b) following marathon running, on markers of post-exercise recovery, in comparison to CHO.

## 2. Materials and Methods

This manuscript comprises data from two separate studies, which utilized nearly identical study designs and methods. For conciseness, a complete description of the methodology of the first study is provided, followed by noteworthy aspects of the second study. Both of the studies were intended as ‘proof of concept’ studies, in order to determine if protein ingestion resulted in any changes in post-marathon recovery, assessed in a field-based protocol. Due to the unique demands of marathon running, a within-subject crossover design was not possible for these studies. As such, both of the studies utilized a parallel-groups pre-post design, in which changes in depending measures (i.e., Δ scores from pre-marathon to post-marathon) were compared between treatment groups (CHO and CP). Subjects in both studies provided consent to participate after receiving written and oral information regarding experimental procedures and potential risks. All of the procedures were approved by James Madison University’s Institutional Review Board.

### 2.1. Study A—Protein Supplementation During Exercise

#### 2.1.1. Subjects

Subjects were males and females that were recruited from a university course at James Madison University, in which they trained for 15 weeks with the goal of completing a 42.2 km marathon run (Thunder Road Marathon, Charlotte, NC, USA), using a program from Trappe and colleagues [24] (See Table 1). All of the potential subjects were novice runners, with no prior marathon experience, who performed sufficient individualized training to complete an 8 km run at the onset of the 15-week training period. Seventeen subjects volunteered for the study; one subject failed to complete all study measurements (prior to group randomization), resulting in 16 subjects. 

#### 2.1.2. Experimental Design

In order to create two experimental groups with relatively similar recovery responses following distance running, the participants were randomized into treatment groups after being stratified into pairs based on changes in serum creatine kinase (CK) levels following their first 29 km training run (week 11 of program). Serum CK levels were assessed from whole blood samples obtained 24 h prior, and 24 h following this training run, using methods described below (see Dependent Measurements). CK was used as a stratification variable in order to minimize between-group variability in post-exercise responses to a distance run, and because it was the only objective recovery measurement that was used in the present study. Specifically, subjects were listed in rank order of CK response (highest to lowest), and were paired with the next individual in the order, unless that individual was a different sex. In that case, the individual was paired with the next same-sex individual in the order, until there were no remaining subjects of the same sex. Due to an uneven number of male/female subjects, this resulted in one mixed-sex pairing, who both ranked low in their post-exercise CK response. Paired subjects were subsequently randomized to separate experimental conditions (CHO_during_ or CP_during_), using the coin-flip method. Individuals in the CHO_during_ group received carbohydrate supplementation throughout the marathon, while the CP_during_ group received carbohydrate + protein supplementation throughout the marathon. Subjects and researchers were blinded to the experimental treatments, as described below (see Experimental Treatments). Following the marathon, both groups consumed minimal protein for at least 2 h post-exercise, via a ‘feedbag’ of foods with low protein content that was provided by the researchers. Ratings of physical and mental fatigue/energy, muscle soreness, and serum CK levels were obtained 48 h prior to the marathon, and at 24 h and 72 h post-marathon. Details regarding the treatments, dietary controls, and dependent measurements are provided below.

#### 2.1.3. Experimental Treatments

Aid stations were provided by the marathon at the following points: 2.5, 6, 10, 16, 19, 22.5, 25.5, 29, 32, 34.5, 37.5, and 40 km. Subjects bypassed these aid stations, and received all fluid/nutrients from the researchers at a point ~100 m past each aid station. At each station, the researchers provided subjects with a 237 mL bottle of water, and an energy gel. The CHO_during_ group received gels containing calories exclusively from carbohydrate (Gu^TM^; 25 g CHO, 0 g protein, 0 g fat, 55 mg sodium, 35 mg potassium per pack), while the CP_during_ group received isocaloric gels containing a mix of carbohydrate and protein (Accel Gel^TM^; 20 g CHO, 5 g protein, 0 g fat, 115 mg sodium, 20 mg potassium per pack). Gel packets were covered with tape, and were the same flavor (orange or vanilla, based on subject preference) to blind the subjects and researchers to their experimental treatment (packets were labelled ‘A’ or ‘B’ by an individual not involved in data analysis, so that the researchers could administer the appropriate treatments without knowledge of their contents). Due to the individual differences in GI tolerances, subjects were not required to consume gels at all of the aid stations, but were encouraged beforehand to consume fluid/gels to their tolerance. All of the subjects received information prior to the event regarding the potential benefits of fluid/carbohydrate ingestion during marathon running (i.e., [23]), and practiced fluid/gel intake during training runs >25 km. The protein content of the CP gels was selected to provide outcomes that were generalizable to marathon runners (i.e., the carbohydrate/protein content was consistent with commercially available products), and because similar ratios of carbohydrate/protein ingestion during exercise have been previously associated with improvements in post-exercise recovery, in comparison to CHO [9]. Researchers collected empty gel packets from all subjects (i.e., by walking/running alongside runners until any accepted gels were completed) to tally the number that was consumed by each runner throughout the marathon. 

#### 2.1.4. Dietary Controls

To minimize the potential influences of post-exercise protein intake, all the subjects received a post-run ‘feedbag’ upon completion of the marathon (in lieu of consuming items provided by the race organizers). The feedbag included a variety of drinks/snacks with <2 g of protein per item, including: carbohydrate-electrolyte drink (Gatorade^®^; 21 g CHO, 0 g protein & fat), water (500 mL; 0 g CHO, protein & fat), banana (27 g CHO, 1.3 g protein, 0.4 g fat), apple (19 g CHO, 0.4 g protein, 0.2 g fat), chewy granola bar (Great Value^TM^ choco chunk; 18 g CHO, 1 g protein, 2 g fat), powdered mini donuts (Hostess Donettes^®^; 16 g CHO, 1 g protein, 6 g fat per 2 donuts), and a rice crispy bar (Kellogg’s^®^; 23 g CHO, <1 g protein, 2.5 g fat). Subjects were permitted to consume as many of these items as they desired, but refrained from consuming other foods/beverages for at least 2 h following the event. Subjects consumed their first post-run meal at the same time (approximately 4 h post-event), but received no further dietary restrictions for the remainder of the study. 

#### 2.1.5. Dependent Measurements 

Subjects completed a series of measurements at three time points: (1) 48 h prior to the marathon, (2) 24 h following the marathon, and (3) 72 h following marathon. Subjects reported to the laboratory at these time-points after an after an overnight fast. In addition, the subjects performed no vigorous exercise (other than the marathon run) for 48 h prior to each measurement, due to a taper in running training (Table 1), and instructions to refrain from other forms of exercise during the experimental period. Upon arrival at the lab, participants completed the measurements below in the order listed. 

Perceived Muscle Soreness: Soreness ratings were obtained using a 100 mm visual analog scale, with 0 indicating no muscle soreness and 100 indicating impaired movement due to muscle soreness, as described previously [8]. Subjects completed this scale immediately upon ascending a flight of 14 steps at normal walking speed without the aid of handrails (Soreness_-A_), and after descending the same flight of stairs without using handrails (Soreness_-D_).

Mental and Physical Energy and Fatigue Ratings: Ratings were obtained using Part II of the Mental and Physical State and Trait Energy and Fatigue Scales (MPSTEFS; P.J. O’Connor, personal communication). Separate ratings were obtained for Physical Energy, Physical Fatigue, Mental Energy, and Mental Fatigue, with subjects being instructed to report “how do you feel right now” at each time-point [8]. Each rating represented the combined scores from three visual analog scales of 0–100 mm (for perceived degree of “energy”, “vigor”, and “pep”), resulting in potential scores of 0–300 mm for each category.

Serum CK: To assess serum CK levels, five mL of venous blood was collected from an antecubital vein after resting in a chair for 15 min. Whole blood was centrifuged at 7000 rpm to separate serum, and stored in a freezer at −80°. Serum CK was subsequently analyzed using a Johnson and Johnson Vitro DT 6011 analyzer, after samples were returned to room temperature. Prior to analyses, the measurement device was calibrated using a reconstituted lyophilized calibration standard purchased from the manufacturer. All of the samples were run in duplicate, and mean values were recorded.

#### 2.1.6. Statistical Analyses

Magnitude-based inferences were utilized to assess the effects of the marathon run and nutrition-group differences on the dependent measures, using the methods described by Hopkins [25]. The threshold for the smallest meaningful treatment effect was quantified as 0.2 × SD (obtained from pre-exercise measurements in the CHO condition) for each variable. Data was analyzed after log-transformation, to diminish the effects of non-uniformity. A published spreadsheet was used to determine the likelihoods of the true treatment effect (of the population), reaching the meaningful change threshold [26]. Likelihoods were classified as: <1% almost certainly no chance, 1–5% = very unlikely, 5–25% = unlikely, 25–75% = possible, 75–95% = likely, 95–99% = very likely, and >99% = most likely. If the likelihood of the effect reaching the threshold was < 25% and the effect was clear, it was classified as a ‘trivial’ effect. If 90% confidence intervals included values that exceeded the threshold for both a positive and negative effect, effects were classified as unclear. As suggested by Hopkins [25], outcomes were not adjusted for multiple comparisons. Data are displayed as raw means ± SD and/or mean difference between treatments ± CL (90% confidence limit).

A published spreadsheet was used to obtain sample size estimates for research designs utilizing magnitude based inferences, which require approximately one-third of the sample size of traditional hypothesis tests [27]. Estimated within- and between-group variances for our dependent measurements (energy/fatigue ratings, soreness, and serum CK levels) were obtained from a prior study in our laboratory, in which these dependent measurements were obtained on multiple occasions from the same group of individuals (*n* = 12), in both the rested state and 24 h after exhaustive exercise (i.e., similar conditions to the present study; unpublished observations). Based on this analysis, our sample size of eight individuals per group had appropriate statistical power (5% chance of type 1 and type 2 clinical errors, using a 90% CL) to detect treatment effects of the following magnitudes: energy/fatigue ratings: 15–22 mm; muscle soreness: 6 mm; serum CK: 32 U/L. 

### 2.2. Study B—Protein Supplementation Following Exercise

#### 2.2.1. Subjects

Subjects were recruited from a subsequent marathon course at James Madison University, one year following Study A. Subjects met the same entry criteria (novice runners with no prior marathon experience), and followed the same training program outlined previously (Table 1). Due to changes in event dates, the subjects completed a different 42.2 km marathon event at the end of their 15-week training program (Rocket City Marathon, Huntsville, AL, USA). Thirteen subjects volunteered for the study; however, injuries unrelated to the study (*n* = 1) and scheduling conflicts (*n* = 2) resulted in three withdrawals prior to data collection. Ten subjects were randomized into one of two treatment groups (described below) using the same approach described previously; i.e., after being paired with another subject who exhibited similar post-exercise changes in serum CK following their first 29 km training run. The only difference in randomization procedures was that no mixed-sex pairs were created, due to an equal number of males/females in the study. One subject was unable to comply with nutritional protocols (did not consume any nutrients in the initial 2 h period post-exercise), so data for this subject and their paired-subject were excluded from data analysis. Therefore, statistical analyses were completed on the remaining eight subjects.

#### 2.2.2. Experimental Design & Nutritional Intervention

The dependent measurements, measurement time-points, and statistical analyses that were used in Study B were identical to the aforementioned protocols for Study A. The only notable difference between studies was the nutritional intervention. The purpose of Study B was to determine the effects of post-exercise protein ingestion on marathon recovery. Therefore, all of the subjects received no protein intake during the marathon; consuming only water, carbohydrate-electrolyte beverages (Powerade^®^; 14 g CHO, 0 g protein & 0 g fat per 240 mL serving), and carbohydrate-electrolyte gels (Gu^TM^; 25 g CHO, 0 g protein, 0 g fat, 55 mg sodium, 35 mg potassium per 32 g pack) that were provided by the event organizers. These items were available at 13 event aid stations at the following distances into the run: 5.4, 7.2, 9.6, 14.4, 18.1, 22.7, 25, 27.5, 30.1, 32.6, 35, 37.3 and 39.7 km. Subjects were permitted to consume items ad libitum from the aid stations, but consumed no other foods/beverages during the marathon.

The two groups received post-exercise feedbags immediately following the marathon, in lieu of foods/beverages from the race organizers. Bags were filled with foods and beverages of similar caloric content, but differing amounts of protein (with resultant offsetting differences in carbohydrate and fat). Feedbags were administered by assistants that were not directly involved with data collection/analysis, to provide blinding to the researchers to the experimental conditions received by each subject. Bags were labeled inconspicuously to provide further blinding to the subjects and researchers to the experimental condition. However, each food/drink item could not be blinded, so it is possible that individuals may have noticed differences in items in their bags versus other subjects. 

The CHO_post_ group (*n* = 4) received a feedbag with a variety of drinks/snacks with low protein content. Specifically, each bag included: 1 carbohydrate-electrolyte drink (Gatorade^®^; 355 mL, 21 g CHO, 0 g protein & fat), 1 chewy granola bar (Great Value^TM^ Choco Chunk; 18 g CHO, 1 g protein, 2 g fat), 1 package of fruit snacks (Welches^®^; 19 g CHO, 1 g protein, 0 g fat), 1 cup of pudding (JELL-O^®^ Chocolate Vanilla Swirls; 14 g CHO, 1 g protein, 1.5 g fat), 1 plain mini-bagel (Thomas’^®^; 24 g CHO, 4 g protein, 1 g fat), 28 g of strawberry jelly (Smuckers^®^; 18 g CHO, 0 g protein, 0 g fat), 1 mini-cake (Little Debbie^®^ Christmas Tree Cake; 28 g CHO, 1 g protein, 10 g fat), 1 rice crispy bar (Kellogg’s^®^; 23 g CHO, <1 g protein, 2.5 g fat), and 1 package of cheddar cheese crackers (Austin^®^; 23 g CHO, 3 g protein, 10 g fat). 

The CP_post_ group (*n* = 4) received a feedbag that included items which contained ≥5 g protein each. The items included were: 1 low-fat chocolate milk (Great Value^TM^; 355 mL, 26 g CHO, 8 g protein, 2.5 g fat), 1 serving of peanut butter pretzels (Utz^®^; 15 g CHO, 5 g protein and 7 g fat), 1 package mixed salted nuts (Planters^®^; 5 g CHO, 6 g protein, 15 g fat), 1 non-fat strawberry Greek yogurt (Chobani^®^; 18 g CHO, 12 g protein, 0 g fat), 1 string cheese stick (Horizon^®^, part-skim mozzarella, 1 g CHO, 8 g protein, 16 g fat), 1 whole-wheat mini-bagel (Thomas’^®^; 22 g CHO, 5 g protein, 1 g fat), 42 g of peanut butter (Smuckers^®^; 10 g CHO, 8 g protein, 22 g fat), and 1 trail-mix chewy granola bar (Kashi^®^ TLC; 20 g CHO, 6 g protein, 5 g fat).

Subjects consumed as many items from their own feedbag as they desired, but refrained from consuming other foods/beverages for at least 2 h following the event. Based on expected post-marathon nutrition intakes (estimated from informal observations following Study A), the foods/beverages in the CP bags were expected to result in average post-exercise protein intakes of ≥20 g, which is consistent with recent recommendations to promote recovery [28]. Following this 2 h period, any food remaining in the feedbags was accounted for, allowing for the researchers to determine each subject’s nutritional intake during the 2-hour period. Subjects consumed their first post-run meal as a group at the same time (approximately 4 h post-event), but received no further dietary restrictions for the remainder of the study. 

Statistical analyses and sample size calculations were performed as described above. Due to a lower recruitment/retention rate in this study, the final sample size (and resultant statistical power) was lower than in Study A [27]. Based on our sample size of four individuals per group, we calculated that we had appropriate power to detect treatment changes of the following magnitudes: energy/fatigue ratings: 25–35 mm; muscle soreness: 9 mm; serum CK: 50 U/L.

## 3. Results

### 3.1. Study A—Protein Supplementation During Exercise

#### 3.1.1. Subjects 

The CHO_during_ group included 5 females and 3 males, with the following baseline characteristics: age = 21 ± 1 y; height = 168 ± 11 cm; weight = 62 ± 9 kg. The CP_during_ group included 6 females and 2 males: age = 20 ± 3 y; height = 167 ± 7 cm; weight = 67 ± 10 kg.

#### 3.1.2. Marathon Outcomes and Nutrient Intake

All of the subjects in Study A completed the marathon run (CHO_during_ = 244.6 ± 35.7 min; CP_during_ = 236.8 ± 26.4 min; no meaningful difference between groups). The CHO_during_ group consumed 4.5 ± 1.4 gels during the run, resulting in 123 ± 36 g CHO ingested (0 g protein, 0 g fat). The CP_during_ group consumed 5.9 ± 1.5 gels, with 118 ± 29 g CHO, 29 ± 7 g protein (0 g fat) during the run. As a result, the protein intake during the marathon was higher in CP_during_ (most likely), and CHO ingestion was similar between groups (unclear).

#### 3.1.3. Dependent Measurements

Mental and Physical Energy/Fatigue Ratings are shown in Table 2. In the CP_during_ group, any changes in these variables from pre-exercise to 24 h returned to baseline levels within 72 h after the marathon. By contrast, the CHO_during_ group exhibited a greater number of meaningful within-treatment effects at 24 h, and some ratings remained elevated 72 h post-exercise. As a result, the likely between-treatment differences were observed for Physical Energy and Mental Fatigue.

**Figure 1:**
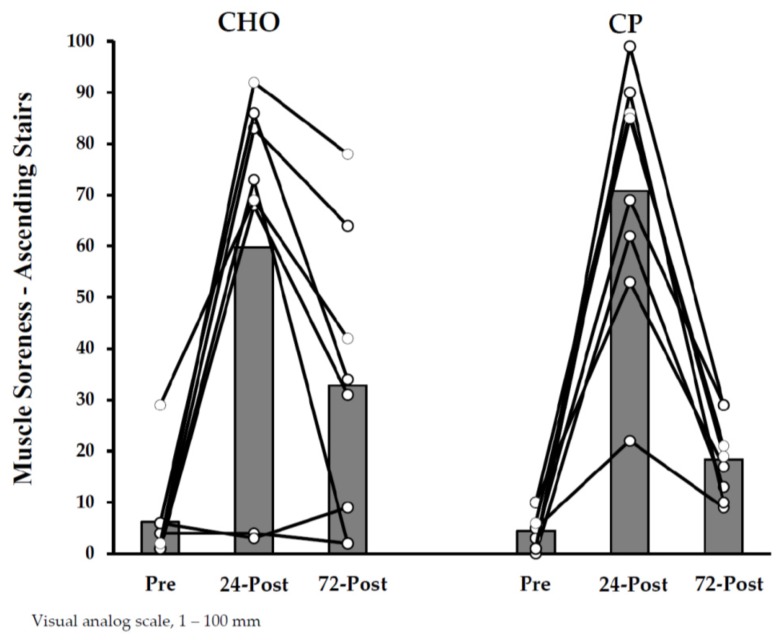
Muscle Soreness Ratings (Ascending Stairs) with Protein Supplementation during Exercise.

**Figure 2:**
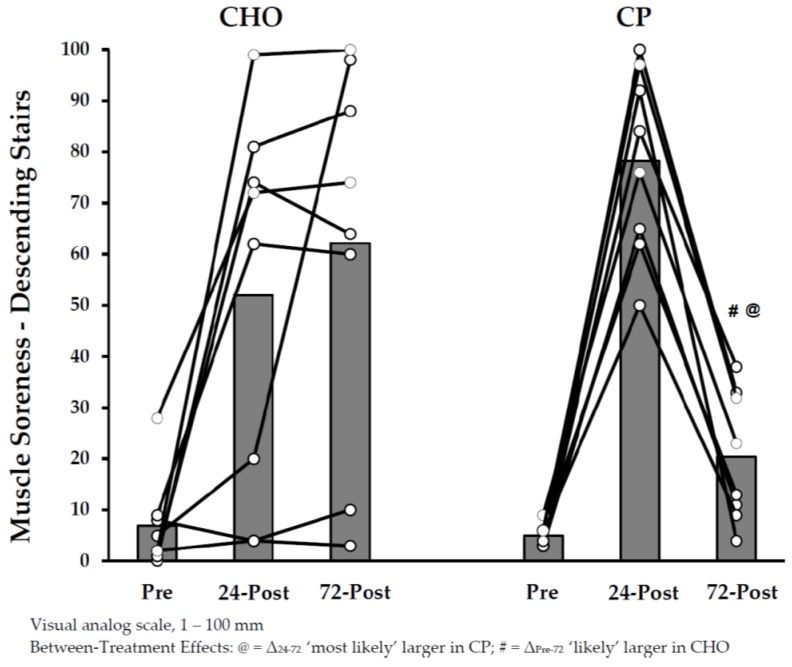
Muscle Soreness Ratings (Descending Stairs) with Protein Supplementation during Exercise.

Muscle soreness ratings are shown in Figure 1 and Figure 2. Soreness increased from pre-exercise to 24 h post-exercise to a similar degree in the groups, with no clear between-treatment effects. Subsequent declines in soreness_-D_ from 24–72 h post-exercise were most likely larger in CP_during_ (versus CHO_during_). As a result, the magnitude to which soreness_-D_ ratings were elevated at 72 h (versus pre-exercise) were likely larger in CHO_during_ than CP_during_.

**Figure 3:**
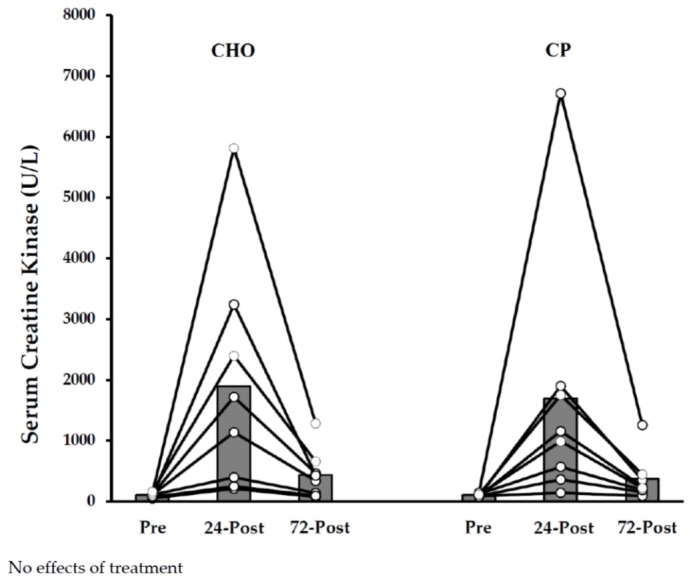
Serum creatine kinase (CK) Responses with Protein Supplementation during Exercise (Study A).

Serum CK values are shown in Figure 3. CK values were most likely increased from pre-exercise to 24 h post-exercise, and remained elevated over baseline levels at 72 h post-exercise. No between-treatment effects were noted at either time-point.

### 3.2. Study B—Protein Supplementation Following Exercise

#### 3.2.1. Subjects 

The CHO_post_ group included two females and two males, with the following baseline characteristics: age = 21 ± 3 years; height = 169 ± 13 cm; weight = 65 ± 12 kg. The CP_post_ group included two females and two males: age = 22 ± 4 years; height = 177 ± 11 cm; weight = 70 ± 13 kg.

#### 3.2.2. Marathon Outcomes and Nutrient Intake

Seven of the eight subjects in Study B completed the marathon run, as one male in the CHO_post_ group was unable to finish. This subject was included in the presented results, as they completed the majority (36 km, ~86%) of the marathon distance. However, analyses were also conducted without this subject (and their respective pair in their CP group), and outcomes from these analyses are also discussed below. The finishing times of the remaining subjects were: CHO_post_ = 230.5 ± 20.5 min; CP_post_ = 253.0 ± 25.8 min (completion times were likely faster in CHO_post_). CHO_post_ consumed 585 ± 229 kcal from their post-exercise feedbag (111 ± 31 g CHO, 5 ± 2 g protein, 13 ± 12 g fat); CP_post_ consumed 489 ± 170 kcal (57 ± 9 g CHO; 28 ± 9 g protein; 17 ± 16 g fat). This resulted in treatment differences in CHO (very likely) and protein (most likely) consumed post-exercise. 

#### 3.2.3. Dependent Measurements

Mental and Physical Energy/Fatigue Ratings are shown in Table 3. Likely-very likely differences between CHO_post_ and CP_post_ were observed in the changes in Physical Fatigue between 24–72 h, and pre-exercise and 72 h. In addition, very likely differences in Mental Energy between groups were observed between pre-exercise and 72 h. 

**Figure 4:**
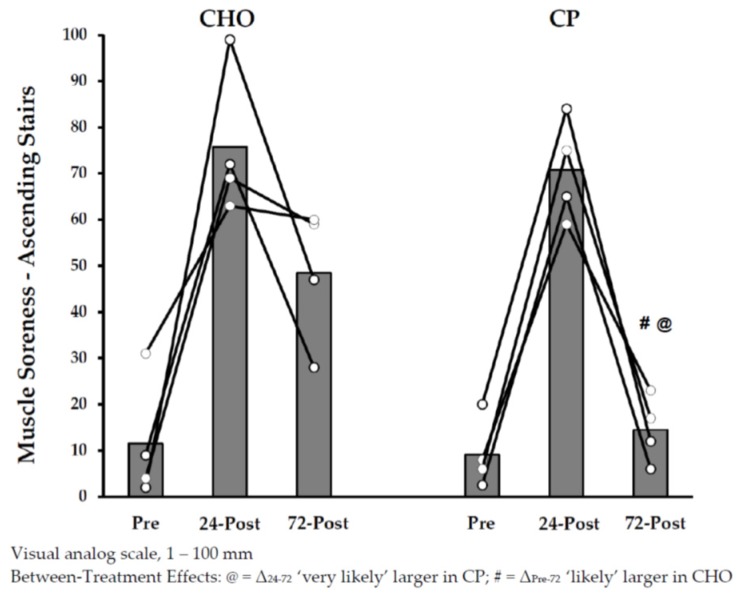
Muscle Soreness Ratings (Ascending Stairs) with Protein Supplementation Post-Exercise.

**Figure 5:**
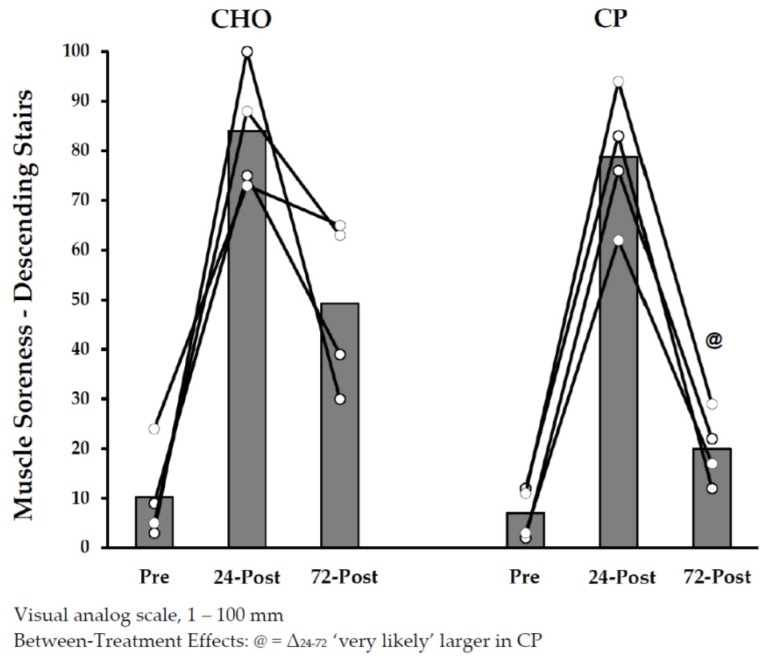
Muscle Soreness Ratings (Descending Stairs) with Protein Supplementation Post-Exercise.

Muscle soreness responses are shown in Figure 4 and Figure 5. Increases in muscle soreness from pre-exercise to 24 h post-exercise were noted in both of the groups, with no clear differences between groups. Soreness ratings declined from 24 h to 72 h post-exercise, with the magnitude of these changes very likely greater in CP_post_ versus CHO_post_. In addition, changes in soreness_-D_ from pre-exercise to 72 h post-exercise were likely greater in CHO_post_ than in CP_post_.

CK values could only be obtained for three subjects in the CHO_post_ group, as one subject was unable to provide blood samples for analysis. As such, the analyses lacked the statistical power to detect any effects which were not very large. Nevertheless, CK values were very likely-most likely increased from pre-exercise to 24 h post-exercise (within-treatments), with no clear differences between groups. CK levels tended to remain elevated over baseline levels at 72 h post-exercise in CP_post_ (99% likelihood) and CP_post_ (93% likelihood), with no meaningful between-treatment effects. 

When data were re-analyzed after removing the CHO subject who did not complete the marathon (and the respective subject from the CP group), the treatment effects/inferences were very similar to those presented for all of the subjects. Specifically, the % likelihoods for any meaningful treatment effects in the entire group were affected in the following manner: mental energy, Δpre-72hr (all subjects = 97% likelihood of benefit with CP; subjects removed = 96% likelihood of benefit with CP); physical fatigue, Δpre-72hr (97%; 91%); physical fatigue, Δ24 hr-72hr (94%; 87%); Soreness_-D,_ Δ24 hr-72hr (96%; 98%), Soreness_-A,_ ΔPre-72hr (91%; 66%); Soreness_-A,_ Δ24 hr-72hr (98%; 92%). In addition, all of the reported unclear treatment effects in the full sample remained unclear after subject removal. 

## 4. Discussion

The primary objective of this study was to assess the effects of CP supplementation on recovery following a marathon run. We observed that CP ingestion during the marathon had no meaningful effects on any recovery markers 24 h post-exercise, in comparison to CHO. However, at 72 h post-marathon, various ratings of soreness and mental and physical energy/fatigue were reduced in CP versus CHO. In addition, the overall directional trends in the aforementioned variables with CP versus CHO were similar when the individuals received CP immediately following the marathon, despite relatively small sample sizes in the CP_post_/CHO_post_ groups. Overall, post-marathon recovery of energy/fatigue/soreness was more complete at 72 h in the groups that received CP, regardless of when they received the nutritional supplementation.

Our observation that CP supplementation was associated with attenuated muscle soreness following exercise has been reported in a number of prior investigations [1,2,3,4,12,29]. Similarly, the enhanced energy/fatigue ratings reported here are consistent with prior reports that CP ingestion is associated with improved post-exercise mood states [3] and possibly diminished tiredness [12]. Our data indicate that subjects who consumed CP during- or post-exercise had improved ratings of soreness/energy/fatigue 72 h following a marathon run, but not 24 h post-exercise. Interestingly, a similar delay in recovery efficacy with CP has been reported in at least two prior studies. Rankin and colleagues [29] reported no meaningful effects of CP (milk, versus an isocaloric CHO beverage) on muscle soreness 24 h after heavy resistance exercise in team-sport athletes. However, at 72 h post-exercise, those who ingested CP had very/most likely lower soreness as compared to CHO. Similar outcomes were also reported for 20 m sprint times, with improved performance with CP noted at 72 h but not 24 h. In addition, Rowlands and colleagues [6] reported that when CP was consumed following an initial 2.5 h bout of cycling, exercise performance (mean power output during repeated-sprints) was not enhanced the following day versus an isocaloric CHO treatment. However, sprint power output in a subsequent performance test (72 h later) was likely to be improved with CP versus CHO. 

The processes involved in recovery following heavy exercise are highly complex, and involve the interaction of metabolic, hormonal, inflammatory, and other factors [28,30]. As a result, it is difficult to ascertain the mechanisms responsible for the efficacy of CP for recovery, and the potential time-course of these changes. The present study was not designed to investigate these mechanisms. However, it is believed that positive muscle protein balance is required to promote the repair of muscle damage and skeletal muscle recovery following heavy exercise [28]. Moore and colleagues [31] have shown that muscle protein synthesis is increased with protein ingestion in a dose-dependent fashion up to ~20 g, with no further increases in protein synthesis beyond this amount. Thus, consuming moderate amounts of protein (~20 g) post-exercise has been recommended to promote protein balance and recovery following heavy exercise [28]. In support of this concept, a series of recent studies have reported that 500 mL of milk ingestion (containing 17 g of protein) was sufficient to promote positive effects on markers of recovery post-exercise, including muscle soreness, CK, and muscle function [7,12,29]. Furthermore, Cockburn [7] reported that 1000 mL of milk provided no further benefits on recovery versus 500 mL. In the present study, measurable improvements in muscle soreness and energy/fatigue after the marathon were observed in the CP_post_ group, who consumed 28 ± 9 g protein post-exercise. These findings provide some evidence that dietary recommendations to consume moderate amounts of protein post-exercise are appropriate for marathon runners to promote recovery. 

The influences of CP consumed during endurance exercise on post-exercise recovery are less extensively studied. In the present study, the CP_during_ group consumed 29 ± 7 g protein during the marathon, which was comparable to the total protein that was consumed by CP_post_. The two groups experienced similar treatment responses in comparison to their respective CHO groups (i.e., no meaningful improvements in recovery at 24 h, enhancements in some ratings of soreness/fatigue/energy at 72 h). It appears from these findings that protein consumption during marathon running has similar effects on post-exercise recovery, in comparison to when protein supplementation is completed post exercise. However, it should be noted that the CP_during_ group consumed addition calories versus CHO_during_ (i.e., equal carbohydrate + additional protein), so the influence of protein ingestion *per se* cannot be ascertained definitively. However, our conclusion that protein intake was responsible for the observed differences in recovery variables is supported by prior evidence from our laboratory. We have previously reported that CP ingestion during exhaustive cycling enhanced post-exercise recovery in comparison to an isocaloric CHO treatment [9]. The present study did not examine whether CP ingestion during and following marathon running augments post-marathon recovery beyond either individual time-point. This finding would strengthen the rationale to recommend protein supplementation during endurance exercise. In support of this, Hall and colleagues [10] reported that CP ingestion during an initial 2.5 h bout of cycling enhanced recovery and subsequent cycling performance (4 h later) versus a CHO trial, even though CP was consumed post-exercise in both trials. However, further investigation is warranted as Breen et al. [16] observed no effects of CP consumed during cycling on similar markers of post-exercise recovery.

Although some studies have reported attenuated post-exercise CK with CP supplementation [2,4,5,6,7,8,9], we observed no clear treatment differences between CP and CHO at 24 h or 72 h. Previously, it has been suggested that CP may be more effective at attenuating less severe muscle damage, following exercise that elicits CK levels of ≤600 U/L [14]. This could have played a role in the outcomes of the present study, as post-marathon CK levels averaged >1000 U/L in all of the groups at 24 h. However, a number of recent studies have reported improvements in the markers of post-exercise recovery (including attenuated CK [7]) following eccentric resistance exercise that elicited higher magnitudes of muscle damage (CK levels) than the present study [7,13,29]. Thus, the reasons for discrepancies in CK responses between studies are presently uncertain. Furthermore, it is not clear why some recovery variables in our study were positively affected at 72 h, but not others. However, this finding is generally representative of the overall literature on this topic, as a majority of the aforementioned studies report that CP ingestion has positive effects on some, but not all, of the recovery variables studied. Further study is necessary to elucidate the reasons for these inconsistencies within- and between-studies. Other factors may have also affected our ability to detect treatment differences in the present study. For example, the sample sizes in the present study were relatively small. As a result, our design lacked the statistical power to detect small, but potentially important treatment effects. This was particularly true in Study B (CP_post_ versus CHO_post_), and specific generalizations based upon this sub-study should be made with caution. Nevertheless, the two sub-studies provide corroborative evidence regarding the potential efficacy of CP supplementation for post-marathon recovery. Another limitation of the present study is that dietary intakes were not controlled prior to the marathon, or between ~6–72 h post-exercise. Thus, differences in pre-marathon nutrient intake could have influenced muscle glycogen levels, and the differences in protein intake throughout the post-exercise period could have contributed variance to the study outcomes. Similarly, carbohydrate/fluid was consumed *ad libitum* during the marathon in Study B, so it is possible that there could have been meaningful differences between groups in the carbohydrate/calories that were consumed during exercise. 

## 5. Conclusions

In conclusion, CP supplementation provided during marathon running did not influence markers of post-exercise recovery at 24 h, but resulted in meaningful reductions in ratings of soreness/energy/fatigue 72 h post-marathon. Furthermore, post-exercise CP supplementation was examined in a smaller sample of subjects, and provided similar outcomes to those that were observed when CP was consumed during exercise (i.e., no treatment effects at 24 h, but improvements in some markers of soreness/energy/fatigue at 72 h). These findings provide evidence that marathon runners derive some recovery benefits from dietary recommendations to consume moderate amounts of protein post-exercise [28], and indicate that further study is warranted to determine the independent and combined effects of CP ingestion during and following heavy endurance exercise.

## Figures and Tables

**Table 1:** Marathon Training Program Showing Distance (km) Completed Per Run.

Week	Monday	Tuesday	Wednesday	Thursday	Friday	Saturday	Sunday	Total
1	5	6	-	5	-	8	-	24
2	5	6	-	5	-	10	-	26
3	5	6	-	5	-	11	-	27
4	5	8	-	5	-	13	-	31
5	5	8	-	5	-	16	-	34
6	6	8	-	6	-	17.5	-	37.5
7	6	10	-	6	-	19	-	41
8	6	10	-	6	-	22.5	-	44.5
9	6	11	-	6	-	25.5	-	48.5
10	8	13	-	8	-	25.5	-	54.5
11	8	13	-	8	-	29	-	58
12	8	13	-	8	-	29	-	58
13	8	13	-	8	-	14.5	-	43.5
14	5	8	-	5	-	13	-	31
15	5	5	-	5 (walk)	-	42.2		57.2

**Table 2:** Energy/Fatigue Ratings with Protein Supplementation during Exercise (Study A).

		Within-Treatment Effects (Mean ± SD)			Between-Treatment Differences Mean ± 90% CI % Likelihoods*, Inference			
Variable	Treatment	Pre	24-Post	72-Post	Pre-24	24–72	Pre-72	
Physical Energy	CHO	240 ± 40	108 ± 64****	166 ± 77	45 ± 79	43 ± 87	88 ± 88	
					76/4/20	75/4/21	94/1/5	
					Unclear	Unclear	Likely positive	
	CP	200 ± 88	113 ± 63	214 ± 64###				
Physical Fatigue	CHO	51 ± 40	191 ± 75****	106 ± 70**,##	−7 ± 90 32/20/48 Unclear	−64 ± 74 5/10/85 Unclear	−71 ± 74 8/9/84 Unclear	
	CP	84 ± 101	217 ± 69****	67 ± 48####				
Mental Energy	CHO	230 ± 56	162 ± 71***	206 ± 76	57 ± 79 72/6/22 Unclear	5 ± 63 52/14/34 Unclear	62 ± 62 76/5/19 Unclear	
	CP	149 ± 94	137 ± 80	186 ± 92##				
Mental Fatigue	CHO	75 ± 73	135 ± 77***	75 ± 72###	−40 ± 93 13/21/66 Unclear	−13 ± 70 21/29/50 Unclear	−53 ± 60 1/10/89 Likely negative	
	CP	147 ± 101	166 ± 78	94 ± 88***,##				

All variables rated on a 0–300 mm scale (the cumulative value from three 100 mm visual analog scales); Within-Treatment Effects: Differences versus Pre values: * = Possible (25–75%), ** = Likely (75–95%), *** = Very likely (95–99%), **** = ML = Most likely (>99%). Differences versus 24-Post values: ## = Likely (75-95%), ### = Very likely (95–99%), #### = ML = Most likely (>99%); CHO = Carbohydrate, CP = Carbohydrate + Protein; * % Likelihoods of positive/trivial/negative effects in the population.

**Table 3:** Muscle Soreness Ratings (Ascending Stairs) with Protein Supplementation Post-Exercise.

		Within-Treatment Effects Mean ± SD			Between-Treatment Differences Mean ± 90% CI% Likelihoods*, Inference		
Variable	Treat	Pre	24-Post	72-Post	Pre-24	24–72	Pre-72
Physical Energy	CHO	233 ± 58	94 ± 63	137 ±38	33 ± 133 76/1/22 Unclear	69 ± 86 29/2/69 Unclear	102 ± 111 93/2/5 Unclear
	CP	202 ± 52	96 ± 35**	207 ± 39###			
Physical Fatigue	CHO	54 ± 68	208 ± 66**	154 ± 27**,##	−49 ± 174 13/6/82 Unclear	−102 ±96 3/2/94 Likely negative	−152 ± 107 2/1/97 Very likely negative
	CP	118 ± 87	223 ± 43	66 ± 61###			
Mental Energy	CHO	216 ± 54	131 ± 60	155 ± 27	56 ± 134 75/5/20 Unclear	42 ± 92 56/10/34 Unclear	98 ± 84 97/1/2 Very likely positive
	CP	184 ± 62	154 ± 57	220 ± 46**			
Mental Fatigue	CHO	91 ± 74	175 ± 40	148 ± 31#	−59 ± 150 20/6/74 Unclear	−35 ± 93 9/9/82 Unclear	−93 ± 100 10/3/87 Unclear
	CP	106 ± 84	131 ± 72	69 ± 59			

All variables rated on a 0–300 mm scale (the cumulative value from three 100 mm visual analog scales); Within-Treatment Effects: Differences versus Pre values: * = Possible (25–75%), ** = Likely (75–95%), *** = Very likely (95–99%), **** = ML = Most likely (>99%). Differences versus 24-Post values: ## = Likely (75–95%), ### = Very likely (95–99%), #### = ML = Most likely (>99%); CHO = Carbohydrate, CP = Carbohydrate + Protein; * % Likelihoods of positive/trivial/negative effects in the population.
